# Thyroid cancer-specific mortality during 2005–2018 in Korea, aftermath of the overdiagnosis issue: a nationwide population-based cohort study

**DOI:** 10.1097/JS9.0000000000001767

**Published:** 2024-06-14

**Authors:** Kyeong Jin Kim, Jimi Choi, Sue K. Park, Young Joo Park, Sin Gon Kim

**Affiliations:** a Department of Internal Medicine, Division of Endocrinology and Metabolism, Korea University College of Medicine Seoul; b Department of Preventive Medicine, Seoul National U College of Medicinea Seoul; c Integrated Major in Innovative Medical Science, Seoul National University College of Medicine Seoul; d Department of Internal Medicine, Seoul National University College of Medicine Seoul; e Department of Molecular Medicine and Biopharmaceutical Sciences, Graduate School of Convergence Science and Technology, Seoul National University, Seoul, South Korea

**Keywords:** mortality trend, thyroid cancer, thyroid cancer-specific mortality

## Abstract

**Background:**

Thyroid cancer (TC) has underwent notable changes in its diagnosis and treatments following the concerns regarding overdiagnosis and overtreatment. However, there is little research on evaluating the effects of these alterations on TC-specific mortality.

**Materials and methods:**

This population-based cohort study included 434 228 patients with TC using Korean National Health Insurance Service-National Health Information Database. The age-standardized and sex-standardized mortality rates of TC per 1000 person-years were calculated considering the number of patients diagnosed with TC in 2013 per our database to evaluate the TC-specific mortality trends according to the year of TC diagnosis.

**Results:**

The authors enrolled 434 228 patients with TC, including 352 678 women and 81 550 men, with a mean age of 48.6±12.5 years and a median follow-up duration of 7.4 (interquartile range: 4.5–10.1) years. TC incidence increased from 2005 to 2012, with a standardized rate of 91.9 per 100 000 people in 2012, decreased rapidly to 50.6 in 2015, and remained stable until 2018. However, TC-specific age-standardized and sex-standardized mortality rates decreased from 1.94 per 1000 person-years in 2005 to 0.76 per 1000 person-years in 2013 and then increased to 2.70 per 1000 person-years in 2018. The TC-specific age-standardized and sex-standardized mortality rates of patients who had undergone hemithyroidectomy or subtotal thyroidectomy remained steady during 2005–2018, but increased in patients who had undergone total thyroidectomy or not undergone thyroidectomy between 2013 and 2018.

**Conclusions:**

The TC-specific mortality rates among patients with TC diagnosed since 2015 have increased, in contrast to the significant decline in TC incidence during the same period. This underscores the importance of appropriate diagnosis and treatment in patients with TC at high-risk of progression, simultaneously emphasizing efforts to reduce overdiagnosis and overtreatment in those with low-risk TC.

## Introduction

Highlights

- The incidence of thyroid cancer (TC) was the highest in 2012, decreased sharply between 2013 and 2015, and then remained stable until 2018 in Korea with 434 228 patients with TC.
- However, the TC-specific mortality was the inversely lowest in 2013 and significantly increased until 2018, especially of patients who underwent total thyroidectomy and did not undergo thyroidectomy.
- Additional research is necessary to evaluate whether the recent increase in TC-specific mortality implies that high-risk TC patients may have been overlooked in Korea.

Thyroid cancer (TC) is the most common malignancy in Korea, with rapidly increasing incidence rates worldwide^1–4^. A sharp rise in TC incidence was observed following the launch of the nationwide cancer screening program in Korea in 1999^2^. Despite TC not being included in the screening program, the widespread utilization of low-cost ultrasonography (US) examinations and advancements in US technology led to a notable increase in TC diagnoses. Ahn *et al*.^2^ raised concerns regarding the ‘overdiagnosis’ of TC, based on the sharp increase in incidence but low disease-specific mortality. This increase, largely driven by small low-risk papillary TC (PTC) has been reported worldwide, including in the United States^5,6^. Accordingly, the social issue of overdiagnosis in thyroid US has been discussed in Korea. Expert recommendations on TC screening have been developed (Level GRADE I: not routinely recommend)^7^, and the importance of adherence for fine-needle aspiration criteria according to the guideline has been emphasized^8^. In line with this, An and Welch^9^ reported a significant decrease in the number of TC operations in South Korea, followed by a continued trend of declining TC incidence.

On the other hand, TC-specific mortality rates in South Korea have been declining steadily from the early 2000s to the late 2010s, even after the sharp decline in incidence in 2014^10–13^. However, these studies have potential limitations as they only analyzed TC-related mortality in the general population in a given year, regardless of the time of initial diagnosis, which may overlook the immediate impact of changes in current diagnostic and treatment modalities. Indeed, little is known about changes in mortality rates for patients diagnosed after the dramatic decline in the incidence of TC in 2014.

Some reports have recently shown an increasing incidence of advanced PTC and PTCs >5 cm in diameter^6,14^. Moreover, survival rates in the screening detection group were significantly higher for advanced differentiated TC than in the clinical detection group^15^, suggesting that prompt management is essential for patients with advanced TC. In addition, survival benefits from early detection of TC have been investigated in comparison with symptomatic TC^16,17^. In this context, the aim of the present study was to investigate recent trends in TC incidence and TC-specific mortality accounting for the calendar year of TC diagnosis in Korea from 2005 to 2018 using the largest retrospective nationwide TC cohort.

## Methods

### Data source and study population

We investigated the trends in TC incidence and TC-specific mortality using the Korean National Health Insurance Service-National Health Information Database (NHIS-2020-1-524), a compulsory medical insurance system that consistently covers for more than 97% of South Korea’s entire population (*n*=52 556 653). The database included longitudinal information on individuals’ demographic, medical, and pharmaceutical data based on the International Classification of Diseases, 10th revision (ICD-10), merged with death records that listed the specific causes of death based on ICD-10 codes and were managed by the Korean National Statistical Office. A more detailed cohort protocol has been published^18,19^.

We included 434 228 patients newly diagnosed with TC (ICD-10 code: C73) between 1 January 2005 and 31 December 2018. Patients with TC were divided into three groups according to their surgical status (Supplementary Fig. 1, Supplemental Digital Content 1, http://links.lww.com/JS9/C779): never thyroidectomy, total thyroidectomy (TT), and less-than-total thyroidectomy (subtotal thyroidectomy and hemithyroidectomy). This study has been reported in line with the strengthening the reporting of cohort, cross-sectional, and case–control studies in surgery (STROCSS) criteria^20^ (Supplemental Digital Content 2, http://links.lww.com/JS9/C780).

### Outcome definition

The follow-up duration was defined as the time from the first date of the TC claim to the date of the death claim or the last data collection in this cohort (31 December 2019). Age-standardized and sex-standardized mortality rates were analyzed to determine TC-specific deaths in patients diagnosed with TC in the indicated year.

### Statistical analysis

Continuous data are expressed as mean±SD for normally distributed variables and median (interquartile range) for non-normally distributed variables. Categorical data are expressed as frequency (percentage). All standardized rates were estimated using the direct standardized method. The incidence rate of TC in South Korea was calculated by dividing the number of newly diagnosed TC cases by the total number of beneficiaries of Korean health insurance each year. Age-standardized and sex-standardization for the incidence rate of TC was estimated based on the age distribution (5-year intervals) and sex distribution of 2013 beneficiaries of Korea’s health insurance. Mortality rates for patients with TC were calculated by dividing the number of deaths by the total person-years of all TC patients and represented as the rate per 1000 person-years (PYs) according to the year of TC diagnosis. To estimate the standardized mortality rates in TC patients, we used the age distribution (5-year intervals) and sex distribution based on person-years of all TC patients in 2013 as reference population in the calculations. The Kaplan–Meier curve was used to compare TC mortality over time among the three groups according to the calendar year of the TC diagnosis (2005–2011, 2012–2014, and 2015–2018). Moreover, we identified changes in mortality rate trends using joint point regression analyses and calculated the annual percentage change (APC) between the trend-change points. The incidence rate ratios and corresponding 95% CI for TC mortality in patients with TC were estimated using a log-linear model, adjusted for age and sex. All analyses were performed using available data, and we did not impute any missing data. All reported *P*-values were two-sided, and statistical significance was set at *P*<0.05. All statistical analyses were performed using SAS Enterprise Guide version 7.1 (SAS Institute Inc.) and R software version 4.1 (R Foundation for Statistical Computing).

## Results

### Baseline characteristics

**Figure 1:**
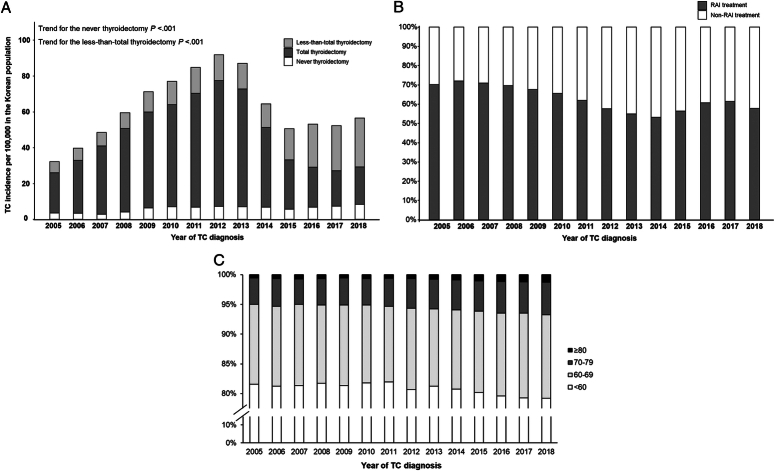
Trends of thyroid cancer (TC) incidence rate in Korean population (A), proportions of radioactive iodine (RAI) therapy among patients undergoing total thyroidectomy in patients with thyroid cancer (TC)(B), and distribution of age at TC diagnosis in patients with thyroid cancer (TC) (C) according to year of TC diagnosis.

Overall, 434 228 patients, including 352 678 women and 81 550 men, were newly diagnosed with TC with a mean age of 48.6±12.5 and a median follow-up of 7.4 (4.5–10.1) years during 2005–2018 in Korea (Table 1). The TC incidence in Korea increased during 2005–2012, with a standardized rate of 91.9 per 100 000 people in 2012, decreased rapidly to 50.6 in 2015, and remained stable from 2015 to 2018 (Fig. 1A). The initial TT was performed in 288 244 (66.4%) patients, less than TT in 103 635 (23.9%) patients. No thyroidectomy was performed in the entire period in 42 349 (9.8%) patients. The proportion of patients who did not undergo any type of thyroidectomy also increased from 11.1% in 2005 to 14.9% in 2018 (*P* for trend <0.001), with the lowest rate of 7.9% in 2012. Of the 391 879 patients who underwent thyroidectomy, the proportion of patients who underwent less-than-total thyroidectomy increased sharply from 2013, accounting for more than half of all surgical procedures performed in 2018 (56.6%; *P* for trend <0.001, 2005–2018). A total of 397 728 (91.6%) patients were prescribed levothyroxine for more than 90 days. Radioactive iodine (RAI) therapy was administered to 44.3% of the patients, and the trend of RAI therapy was lowest in 2014 and then slightly increased among patients with TT (Fig. 1B). Supplementary Table 1 (Supplemental Digital Content 1, http://links.lww.com/JS9/C779) and Fig. 1C indicate that the age of patients aged 70 years at the TC diagnosis consistently increased between 2005 and 2018.

**Table 1:** Baseline characteristics of patients with thyroid cancer.

	Patients with thyroid cancer
Mean±SD or *n* (%)	*n*=434 228
Age (years)	48.6±12.5
Female	352 678 (81.2)
Surgical treatment	
Never thyroidectomy	42 349 (9.8)
Less-than-total thyroidectomy	103 635 (23.9)
Total thyroidectomy	288 244 (66.4)
Levothyroxine	397 728 (91.6)
RAI	192 451 (44.3)
Follow-up duration (years), median (IQR)	7.4 (4.5–10.1)
BMI (kg/m^2^)	24.0±3.4
SES	
Low (1st tertile)	91 586 (21.1)
Middle (2nd tertile)	127 922 (29.5)
Upper (3rd tertile)	214 720 (49.4)
Smoking	
Unknown	102 750 (23.7)
Never	276 822 (63.8)
Ex-smoker	26 380 (6.1)
Current	28 276 (6.5)
Alcohol consumption	
Unknown	102 368 (23.6)
Never	227 506 (52.4)
≤2 times a week	84 463 (19.5)
≥3 times a week	19 891 (4.6)
Comorbidities	
Hypertension	149 017 (34.3)
Diabetes mellitus	41 641 (9.6)
Dyslipidemia	110 773 (25.5)

IQR, interquartile range; RAI, radioactive iodine treatment; SES, socioeconomic status.

### Trends of TC-specific mortality

**Figure 2:**
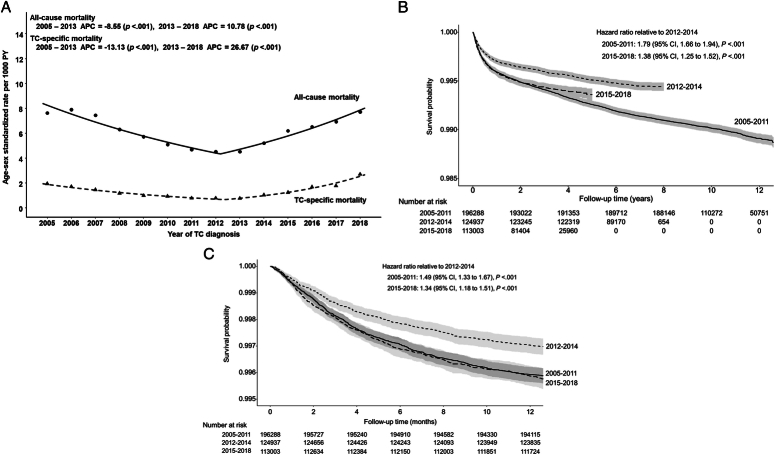
Annual percentage changes (APC) in age-standardized and sex-standardized overall and TC-specific mortality rates in patients with thyroid cancer (TC) according to the diagnosis in the indicated year. (A). Kaplan–Meier survival analysis of TC-specific mortality according to the TC diagnosis period during the whole period (B) and TC-specific mortality within 1 year after TC diagnosis (C) of patients with thyroid cancer (TC).

The age-standardized and sex-standardized mortality rate in patients with TC diagnosed in the indicated year decreased from 1.94 per 1000 PY in 2005 to 0.76 per 1000 PY in 2013 and then increased to 2.70 per 1000 PY in 2018 (Fig. 2A). This trend was inversely related to the incidence of TC trend. The APC for TC-specific standardized mortality differed significantly with –13.13 (*P*<0.001) during 2005–2013 and 26.67 (*P*<0.001) during 2013–2018 (Fig. 2A). The Kaplan–Meier survival analysis showed similar patterns of TC-specific mortality according to the TC diagnosis period (Fig. 2B). Patients with TC diagnosed between 2005 and 2011 had the lowest survival rates, followed by those diagnosed between 2015 and 2018 and 2012 and 2014, who had high survival rates. The mortality rate within 1 year after the TC diagnosis, which can be assumed to be an advanced stage of TC, was the lowest for patients diagnosed during 2012–2014, and similarly higher patterns were observed during 2005–2011 and 2015–2018 (Fig. 2C). This suggests that since 2015, the number of patients dying from TC who had already advanced at the time of diagnosis has increased. Therefore, we grouped patients by the year of TC diagnosis and analyzed mortality during the follow-up in subsequent analyses.

**Figure 3:**
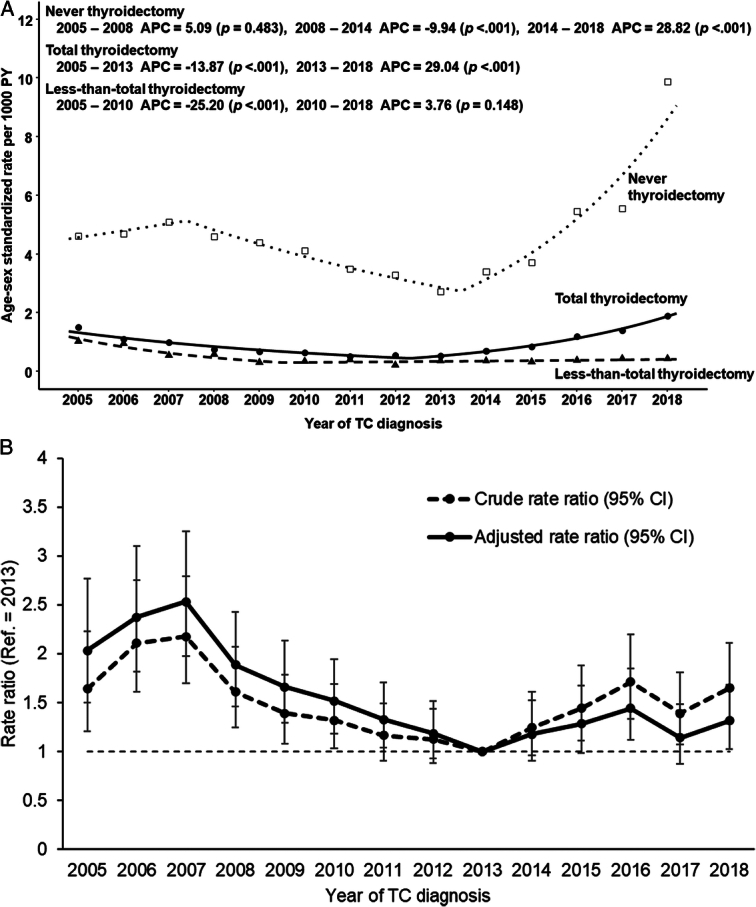
Mortality trends according to the surgical treatment status (A). and crude, age-adjusted, and sex-adjusted rate ratios for TC-specific mortality as references for patients diagnosed in 2013 in patients with thyroid cancer (TC) (B).

For thyroidectomy subtypes, TC-specific mortality of patients who underwent less-than-TT remained steady from 2005 to 2018; however, for those who underwent TT, and particularly those with never thyroidectomy, TC-specific mortality increased from 2013 to 2018 (Fig. 3A). The crude and age-adjusted and sex-adjusted rate ratios for TC-specific mortality, as a reference for patients diagnosed in 2013, were significantly higher in patients diagnosed between 2014 and 2018 (Fig. 3B).

**Figure 4:**
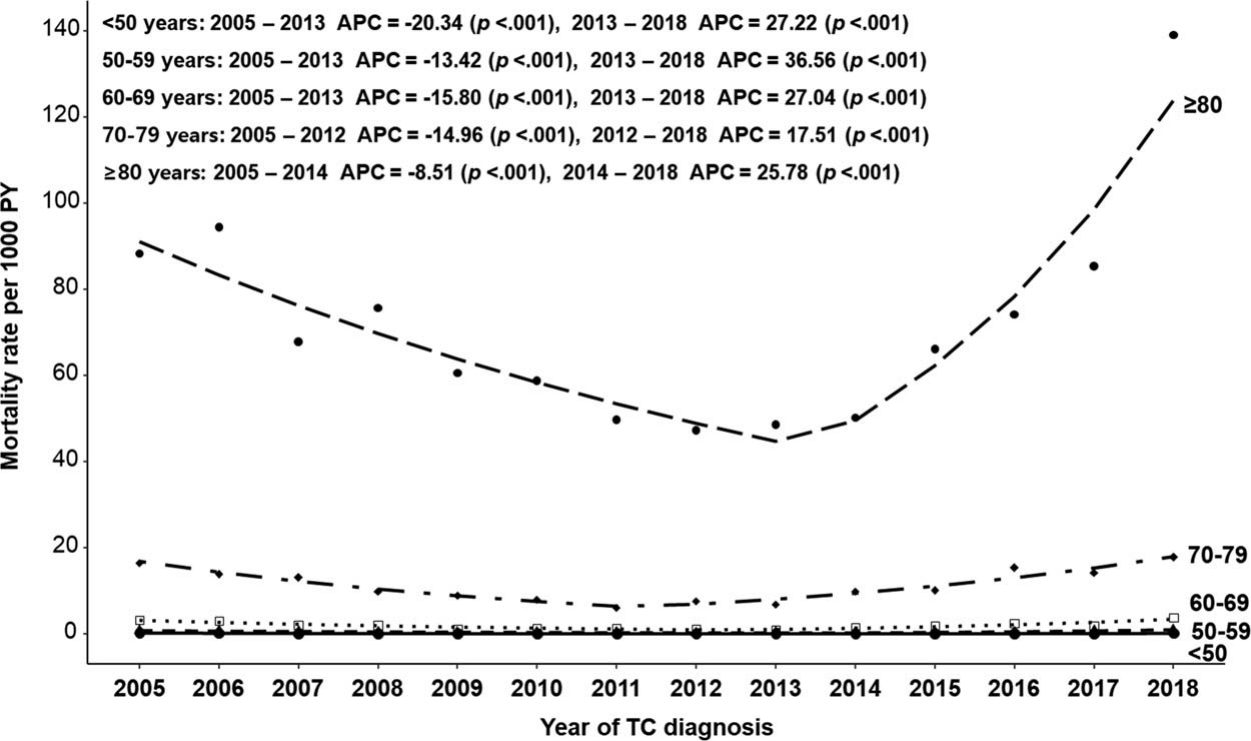
Annual percentage changes (APC) in thyroid cancer (TC)-specific mortality rates per 1000 person-years by age group.

Figure 4 shows the APCs of TC-specific mortality rates according to age at the TC diagnosis. TC-specific mortality increased with age, and the trend in TC-specific mortality APCs, which showed a decreasing pattern from 2005 to 2012 (or 2013) and then increasing until 2018, was similar across all age groups, but a robust increase was seen in those aged 80 or older.

## Discussion

TC has initiated a global debate regarding overdiagnosis and overtreatment, which have reduced the incidence of TC in the USA as well as South Korea^21,22^. Consequently, in recent years, there have been concerns about the trend in TC-specific mortality rates subsequent to a sharp decline in incidence following an abrupt surge in TC incidence. In this context, our results demonstrated that the TC-specific mortality trend has increased since 2013, when the incidence of TC was the highest, using the largest nationwide TC cohort in Korea, which is known for the most rapid increase in TC incidence. Moreover, a robust increase was observed in elderly patients and those with no surgical treatment or TT.

The subsequent increase in mortality rates since 2013 has raised concerns and necessitates further long-term investigations. The substantial increase in TC incidence in Korea is mainly because of the increased incidence of low-risk PTC^23,24^ and the revised guidelines, such as the guidelines of the less aggressive Korean Thyroid Imaging Reporting and Data System (K-TIRADS)^25^, American Thyroid Association^4^, and Korean Thyroid Association^8^, may have contributed to the decline in low-risk PTC incidence, modifications in the scope of surgical thyroidectomy, and increase in active surveillance (AS) for low-risk PTC. Although the alteration in guidelines for diagnosing and treating low-risk TC is unlikely to directly contribute to increased mortality, the rise in mortality among older patients or those who have undergone total thyroidectomy implies a growing number of diagnoses of late-stage or incurable conditions, leaving the underlying reasons for this trend to be elucidated. These changes have contributed to the recent increase in less-than-TT and never thyroidectomy rates, challenging the prevalent tides of overdiagnosis and overtreatment in South Korea. The present study found that the APC for TC-specific mortality increased only in patients who underwent TT or never thyroidectomy but not in those who underwent less-than-total thyroidectomy. Patient age at diagnosis has been widely used as a major mortality risk factor, with elderly patients often presenting with more advanced disease stages and poorer prognoses. And the absence of surgical intervention may suggest either a diagnosis at a stage where surgical treatment was deemed not beneficial or the presence of comorbid health conditions that made surgery difficult to perform. However, due to the lack of pathological information in our database, it is challenging to definitely attribute the increased mortality rates among TT and never-thyroidectomy patients to a rise in the incidence of aggressive TC. Furthermore, the mortality has increased in recent years in all age groups, and not just in older patients. Therefore, the observed rise in TC-specific mortality rates among patients recently diagnosed with TC may suggest the potential under-recognition of high-risk TC patients, necessitating further dedicated investigation. In the current context, it is imperative to ensure precise evaluation of cervical lymph nodes in patients with thyroid nodules and maintain adherence to accurate indications for AS. A few can growth very rapidly in low-risk PTCs^26^, suggesting that regular follow-up should be performed, and the development in molecular markers or artificial intelligence algorithm to identify those with high-risk of progression would be very helpful. Particularly, molecular panel tests can be very informative, but the development of less-expensive tests is anticipated to facilitate their global application outside the USA^27^.

Previous Korean studies that used data from Korean statistics demonstrated that age-standardized mortality rates from TC per 100 000 population increased from 1985 to 2004 and then continuously decreased until 2015^9^. The recent mortality trends were discordant with the present study because the previous study examined the age-standardized mortality rate of TC in that year based on the overall general population, whereas our study analyzed the mortality trend based on the calendar year of diagnosis as a reference for patients with TC diagnosed in 2013.

Megwalu and Moon^28^ reported that TC incidence rates have declined since 2014; however, incidence-based mortality rates have continued to increase using the Surveillance, Epidemiology, and End Results-18 cancer registry in the United States, suggesting an underlying true increase in TC incidence that is not completely accounted for by the overdiagnosis of small indolent tumors. Korea, which showed a robust increase and decrease in TC incidence, showed opposite trends in TC-specific mortality and incidence.

The primary strengths of this study include its population-based design, sufficient sample size, accurate detection of mortality, as managed by the Korean National Statistical Office, and minimal loss to follow-up. Moreover, we analyzed the age-standardized and sex-standardized mortality rates of patients with TC according to the year of initial TC diagnosis, which can better reflect the trend of death according to the year of diagnosis and current status.

This study has several limitations. In the NHIS-NHIS database, there is no information on clinicopathological characteristics, such as the pathological subtype and stage, including poorly differentiated TC, anaplastic TC, and medullary TC, which may also be present in all groups, particularly in patients who did not undergo any surgical treatment. However, most TC cases in Korea are DTC, particularly PTC, which accounts for up to 97% of all TC cases, as reported in previous studies. Another major limitation was the lack of biochemical laboratory results for thyroid-stimulating hormone, thyroglobulin antigen, and antibodies that can predict TC disease status. The relatively short follow-up duration is also a major disadvantage of this study. Finally, it is imperative to acknowledge that the heterogeneity of healthcare systems across countries precludes the generalization of our findings to a global trend.

## Conclusion

In summary, the incidence of TC with its highest rate in Korea has sharply decreased since 2012; however, the TC-specific mortality rate has turned to increase since 2013 in the exact opposite direction. Therefore, further research is warranted to investigate whether the recent increase in TC-specific mortality can be attributed to delayed diagnosis in high-risk patients relative to indolent low-risk patients.

## Ethical approval

The study protocol was approved by the Institutional Review Board of the Korea University Anam Hospital (approval number: 2020AN0310).

## Consent

Informed consent was not required because the study was based on data from the NHIS database, which was fully anonymized and de-identified for analyses.

## Source of funding

This research was supported by a grant of Patient-Centered Clinical Research Coordinating Center funded by the Ministry of Health and Welfare, Republic of Korea (grant number: HI19C0481, HC19C0103).

## Author contribution

Y.J.P. and S.G.K.: concept and design; K.J.K., J.C., S.G.P., Y.J.P., and S.G.K.: acquisition, analysis, or interpretation of data; K.J.K.: drafting of the manuscript; J.C.: statistical analysis; K.J.K. and J.C.: administrative, technical, or material support; Y.J.P. and S.G.K.: supervision.

## Conflicts of interest disclosure

No competing financial interests exist for any authors.

## Research registration unique identifying number (UIN)

This study is registered at Clinical Research Information Service KCT0009197; https://cris.nih.go.kr/cris/search/detailSearch.do?search_lang=E&focus=reset_12&search_page=NU&pageSize=10&page=undefined&seq=26623&status=5&seq_group=26623.

## Guarantor

Sin Gon Kim, Korea University College of Medicine, 73 Goryeodae-ro, Seongbuk-gu, Seoul 02841, South Korea. E-mail: k50367@korea.ac.kr.

## Data availability statement

The data that support the findings of this study are available from the cohort committees and national registers of the cohorts and countries involved (the Korean National Health Insurance Service). Restrictions apply to the availability of these data, which were used under license for this study.

## Provenance and peer review

Not commissioned, externally peer-reviewed.

## Supplementary Material
