# Supplementary material for: Thyroid cancer-specific mortality during 2005–2018 in Korea, aftermath of the overdiagnosis issue: a nationwide population-based cohort study
Source: Int J Surg. 2024 Jun 14;110(9):5489–95. doi: 10.1097/JS9.0000000000001767 (PMC11392158; doi:10.1097/JS9.0000000000001767)

**Supplementary Data**

***Kim KJ, Choi J, Park SK et al.* Thyroid cancer-specific mortality during 2005–2018 in Korea, aftermath of the overdiagnosis issue: a nationwide population-based cohort study**

**Supplementary Table 1.** Trends in age at thyroid cancer diagnosis.

**Supplementary Figure 1.** Schematic study design.

**Supplementary Table 1.** Trends in age at thyroid cancer diagnosis.

| Age at TC diagnosis,  n (%), years | 2005 | 2006 | 2007 | 2008 | 2009 | 2010 | 2011 | 2012 | 2013 | 2014 | 2015 | 2016 | 2017 | 2018 |
| --- | --- | --- | --- | --- | --- | --- | --- | --- | --- | --- | --- | --- | --- | --- |
| <20 | 100  (0.7) | 103  (0.6) | 111  (0.5) | 133  (0.5) | 143  (0.4) | 160  (0.4) | 172  (0.4) | 173  (0.4) | 178  (0.4) | 193  (0.6) | 162  (0.6) | 158  (0.6) | 167  (0.6) | 151  (0.5) |
| 20-29 | 922  (6.5) | 1,005  (5.7) | 1,125  (5.1) | 1,456  (5.2) | 1,665  (4.9) | 1,823  (4.8) | 1,910  (4.5) | 2,074  (4.5) | 2,108  (4.7) | 1,794  (5.3) | 1,471  (5.5) | 1,756  (6.2) | 1,755  (6.3) | 2,021  (6.7) |
| 30-39 | 2,648 (18.6) | 3,329 (18.7) | 4,085 (18.4) | 5,133 (18.4) | 6,446 (18.9) | 7,033 (18.7) | 7,775 (18.4) | 8,429 (18.1) | 8,175 (18.3) | 6,068  (18) | 4,992 (18.7) | 5,283 (18.7) | 5,419 (19.4) | 6,045  (20) |
| 40-49 | 4557  (32) | 5,560 (31.3) | 7,063 (31.8) | 8,717 (31.2) | 10,374 (30.4) | 11,224 (29.8) | 12,342 (29.2) | 12,955 (27.9) | 12,909 (28.8) | 9,383 (27.9) | 7,534 (28.3) | 7,825 (27.7) | 7,617 (27.3) | 8,095 (26.8) |
| 50-59 | 3,394 (23.8) | 4,442  (25) | 5,692 (25.6) | 7,388 (26.5) | 9,140 (26.8) | 10,566 (28.1) | 12,484 (29.5) | 13,902 (29.9) | 13,007  (29) | 9,730 (28.9) | 7,225 (27.1) | 7,445 (26.4) | 7,196 (25.8) | 7,597 (25.2) |
| 60-69 | 1,912 (13.4) | 2,390 (13.4) | 3,037 (13.7) | 3,678 (13.2) | 4,623 (13.5) | 4,948 (13.1) | 5,406 (12.8) | 6,345 (13.6) | 5,837  (13) | 4,481 (13.3) | 3,637 (13.6) | 3,929 (13.9) | 3,980 (14.2) | 4,240  (14) |
| 70-79 | 634  (4.5) | 826  (4.6) | 947  (4.3) | 1,243  (4.5) | 1,551  (4.5) | 1,665  (4.4) | 1,985  (4.7) | 2,314  (5) | 2,235  (5) | 1,713  (5.1) | 1,360  (5.1) | 1,495  (5.3) | 1,464  (5.2) | 1,641  (5.4) |
| ≥80 | 75  (0.5) | 119  (0.7) | 157  (0.7) | 178  (0.6) | 190  (0.6) | 247  (0.7) | 257  (0.6) | 308  (0.7) | 337  (0.8) | 289  (0.9) | 281  (1.1) | 334  (1.2) | 340  (1.2) | 388  (1.3) |

**Supplementary Figure 1.** Schematic study design.


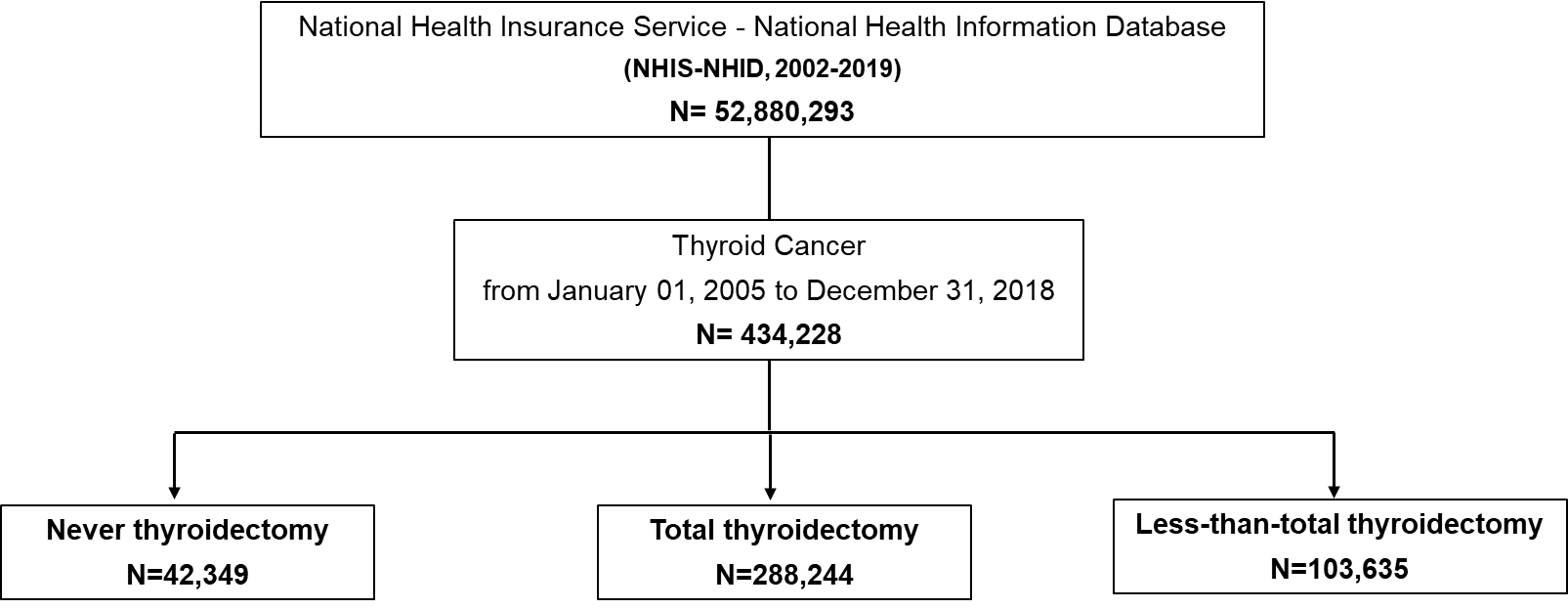

Supplement: Supplementary file 1 [file js9-110-5489-s001.docx]
